# Avoidant authority: The effect of organizational power on decision-making in high-uncertainty situations

**DOI:** 10.3389/fpsyg.2022.1027108

**Published:** 2023-02-24

**Authors:** Neil D. Shortland, Maureen E. McCusker, Laurence Alison, Nikki Blacksmith, Matthew P. Crayne, Lisa Thompson, Joseph Gonzales, Presley McGarry, Catherine Stevens

**Affiliations:** ^1^School of Criminology and Justice Studies, University of Massachusetts Lowell, Lowell, MA, United States; ^2^Institutional Research and Decision Support, Virginia Commonwealth University, Richmond, VA, United States; ^3^Institute for Risk and Uncertainty, University of Liverpool, Liverpool, United Kingdom; ^4^School of Business, University at Albany, Albany, NY, United States; ^5^Department of Management, American University, Washington, DC, United States; ^6^Department of Psychology, University of Massachusetts Lowell, Lowell, MA, United States

**Keywords:** least-worst decision, social power, redundant deliberation, organizational culture, approach/avoid

## Abstract

Individuals in positions of power are often required to make high-stakes decisions. The approach-inhibition theory of social power holds that elevated power activates approach-related tendencies, leading to decisiveness and action orientation. However, naturalistic decision-making research has often reported that increased power often has the opposite effect and causes more avoidant decision-making. To investigate the potential activation of avoidance-related tendencies in response to elevated power, this study employed an immersive scenario-based battery of least-worst decisions (the Least-Worst Uncertain Choice Inventory for Emergency Responses; LUCIFER) with members of the United States Armed Forces. In line with previous naturalistic decision-making research on the effect of power, this research found that in conditions of higher power, individuals found decisions more difficult and were more likely to make an avoidant choice. Furthermore, this effect was more pronounced in domain-specific decisions for which the individual had experience. These findings expand our understanding of when, and in what contexts, power leads to approach vs. avoidant tendencies, as well as demonstrate the benefits of bridging methodological divides that exist between “in the lab” and “in the field” when studying high-uncertainty decision-making.

## Introduction

In the face of multiple, unappealing options, individuals often must commit to courses of action that are less than ideal (Alison et al., 2013). Such decisions can manifest in a range of situations and across a range of organizational levels (Klein, 1993), and include deciding how, if, and when to lockdown a country in response to a global pandemic, or to how to deal with a potentially hostile civilian in a police or military encounter. These decisions all involve multiple potentially negative courses of action, and often have significant, long-lasting implications for the decision-maker, those involved, and society at large (Shortland et al., 2019). Theories of decision-making often center on the assumption that the decision-maker chooses the course of action by identifying the “best” or the choice with the highest “expected value” (Simon, 1955, 1978; Tversky and Kahneman, 1974; Kahneman and Tversky, 1979; Hastie and Dawes, 2009). Yet in many cases, decision makers are presented with courses of action that have uncertain outcomes and in which each potential outcome could have negative implications (Klein, 1993; Cannon-Bowers and Salas, 1998). Such decisions violate traditional theories of decision-making because a “best” course of action cannot be identified (Shortland et al., 2018). Psychologists who study high-uncertainty decision-making in the field refer to these kinds of decisions as “least-worst” in that the decision-maker must calculate and choose the option that causes the least harm from a series of harmful options (Power and Alison, 2017).

Least-worst decisions require the decision-maker to take decisive action and overcome fear, doubt, and uncertainty to commit to a least-worst choice (Alison et al., 2018; Shortland et al., 2019). However, research has indicated that the process of least-worst decision-making can often become derailed, resulting in decision inertia. There are several manifestations of decision inertia, all of which involve the failure to make a necessary decision in time, or the failure to make any decision at all (van den Heuvel et al., 2012). Decision-makers can avoid the choice (decision avoidance; Anderson, 2003), cognitively ruminate over the options for no overall gain (redundant deliberation; Shortland and Alison, 2020), or fail to implement a chosen course of action (implementation failure; van den Heuvel et al., 2012). Decision inertia often stems from decision-makers becoming trapped between an approach-based choice that maximizes progress toward task accomplishment and an avoidance-based choice that minimizes any possible or further harm (Power and Alison, 2017; Power, 2018). Research has begun to explore the environmental antecedents of decision inertia (van den Heuvel et al., 2012; Power and Alison, 2017) as well as individual differences in susceptibility to decision inertia (Shortland et al., 2019, 2020a,2020b; Shortland and Alison, 2020).

One consistent finding in the field is that individuals in positions of power are at a greater risk of redundant deliberation (Alison et al., 2015a,b; Power and Alison, 2017; Shortland et al., 2019). Naturalistic decision-making research has found that, when operating within organizational settings, individuals who hold positions of power avoid committing to a course of action when they anticipate negative consequences because of fears of accountability (Waring et al., 2013) and negative feedback (Eyre et al., 2008), especially when the individual cannot fully justify their choice (Brooks, 2011). From a theoretical standpoint, however, such findings are especially interesting because they run contrary to the body of research that shows that positions of power improve decision-making by removing inhibitions and encouraging action (e.g., Maner et al., 2007, 2010; Smith and Bargh, 2008).

In organizational settings, power is defined as the ability to control resources, one’s own and others,’ without social interference (Galinsky et al., 2003). Power is also defined as the ability to influence another person to do something they would not do without the presence of such power (Hashemian et al., 2019). Accordingly, individuals who hold higher status (rank) in an organization are guaranteed power due to legitimacy (French and Raven, 1959; Boksem et al., 2012), especially in high-stakes situations, because of the control of others, resources, and fewer social barriers to action. It is argued that having power allows the individual to operate with more free will (i.e., less inhibition; Weber, 1914). Power increases approach motivations and assists in the process of goal setting and action prioritization (Cho and Keltner, 2020) by allowing the decision-maker to focus on the task (or goal) at hand (Smith and Bargh, 2008; DeWall et al., 2011). Guinote (2007) found that those with higher power were faster decision-makers and faster to initiate goal pursuit. Cho and Keltner (2020) also found that those with power make faster decisions and act more promptly. Powerful individuals also engage in less deliberate decision-making processes (Fiske and Dépret, 1996) and have less foresight (consideration of “what happens if”; Guinote and Kim, 2020). Power works throughout the entire motivational process, from increasing goal setting, to early action, through to completion of the task (Guinote, 2007). Power is also especially beneficial under pressure with high stakes (Kang et al., 2015). Pike and Galinsky (2020) surmised that power releases the psychological brakes on action by (1) making failure seem less probable and feel less painful (2) decreasing the “downsides” of action, (3) shrouding the feelings and thoughts of others, (4) diminishing the perceived social costs of action, and (5) increasing greater goal focus by limiting goal-inhibiting distractions and focusing the mind on action.

From a theoretical standpoint, power causes activation of the behavioral activation system (BAS; Keltner et al., 2003; Cho and Keltner, 2020). The BAS is the main driving force of approach behavior within Gray’s (1987) Reinforcement Sensitivity Theory (RST). Within the RST framework, stimuli perceived as positive activate the Behavioral Activation System (BAS) and approach behaviors toward target goals, while stimuli perceived as negative activate the Fight, Flight, Freeze System (FFFS), motivating the individual to avoid potential threats. The Behavioral Inhibition System (BIS) coordinates the response by attempting to resolve conflicting inputs when a stimulus activates both BAS and FFFS (McNaughton and Gray, 2000). RST provides a model of how animals, including humans, respond to motivationally significant (i.e., “reinforcing”) stimuli, and how this motivation is mediated by the neuropsychological activity (Corr and Cooper, 2016). Power is often linked to BAS activation because research has found that power increases a generalized approach orientation (Keltner et al., 2003; Cho and Keltner, 2020), optimism and confidence (Fast et al., 2012); and power increases disinhibited behavior (Gonzaga et al., 2008). Furthermore, Krupić and Corr (2017) posit that BAS processes are associated with a “fast lifestyle,” typified by bold, aggressive, and impulsive behavior (Wolf et al., 2007). This has led to the approach-inhibition theory of power, in which “elevated power (which relates to increased rewards and freedom) activates approach-related tendencies, whereas reduced power (which relates to increased threat, punishment, and social constraint) activates inhibition-related tendencies” (Cho and Keltner, 2020, p. 196). Contrary to the approach-inhibition theory of power, the naturalistic decision-making research outlined above implies that in high-uncertainty, blame-centric environments power increases avoidance. As such, rather than BAS activation, power increases FFFS activation, leading to FFFS activation, or become stuck deciding between approach and avoidant courses of action (a failure of BIS to reconcile concurrent FFFS and BAS activation). FFFS/BIS activation is associated with risk-assessment and can be an adaptive process of caution and weighing up all the possibilities (Perkins and Corr, 2006). However, FFFS/BIS activation can also decrease performance by increasing doubt, indecision, worry, and engagement of time-wasting “displacement activities” (Corr et al., 2016), and therefore decision inertia (Alison et al., 2013).

FFFS/BIS activation is driven by fear and anxiety and is viable to propose that the dissociation of findings between laboratory-based research and naturalistic decision-making research is likely driven by the lack of context and accountability in laboratory research paradigms. While laboratory studies often rely on minor tasks with little to no accountability or consequence, naturalistic decision-making research observes high-uncertainty decisions in contexts in which the outcomes of a decision could be grave and the individual is accountable for their actions (Alison et al., 2013; Waring et al., 2013). Such situations are far more likely to evoke FFFS/BIS-associated feelings of fear, anxiety, and self-evaluation (McNaughton and Corr, 2004; Corr et al., 2016). What this means, therefore, is that while the approach-inhibition theory of power may be appropriate in low-stakes environments, it may not apply to those contexts in which power is associated with blame and accountability. Instead, in such contexts there is significant warrant to propose that power can cause FFFS/BIS activation and concurrent tendencies to either (1) engage in avoidant behavior and/or (2) become inert and trapped between approach/avoid motivations.

### Research questions

This study is interested in the degree to which being put in a position of power causes tendencies to take approach-orientated actions. To test this research question, the present study conducts a controlled experimental test of power and decision-making using an ecologically valid inventory of high-uncertainty least-worst decisions with a sample of senior decision-makers. Specifically, we hypothesize that:

H_1_: High power is positively related to the speed it takes to make a least-worst decision.

H_2_: High power is positively related to perceived difficulty of making a least-worst decision.

H_3_: High power is negatively related to the tendency to make approach-orientated least-worst decisions.

## Materials and methods

### Participants

A total sample of 234 United States (U.S.) Army soldiers, including both officers and enlisted, across a range of ranks completed this study (77.78% male, age: *M* = 31.81 years, *SD* = 5.99; 61.54% had been previous deployed to war overseas) from a total of three U.S. Army bases across the nation. Recruitment for this study was completed in support of a wider study on individual differences in decision-making conducted in support of the Foundational Science Research Unit (FSRU) U.S. Army Research Institute (ARI). Data were collected using Qualtrics (Provo, UT) software installed on individual iPad tablets provided to the individual soldiers. Two members of the research team administered the assessments to the soldiers, ensuring adequate distance among the soldiers for privacy in completing the assessments. The entire assessment took approximately 45 min to complete.

### Procedure

#### Decision-making scenarios (least-worst uncertain choice inventory for emergency responses)

**FIGURE 1:**
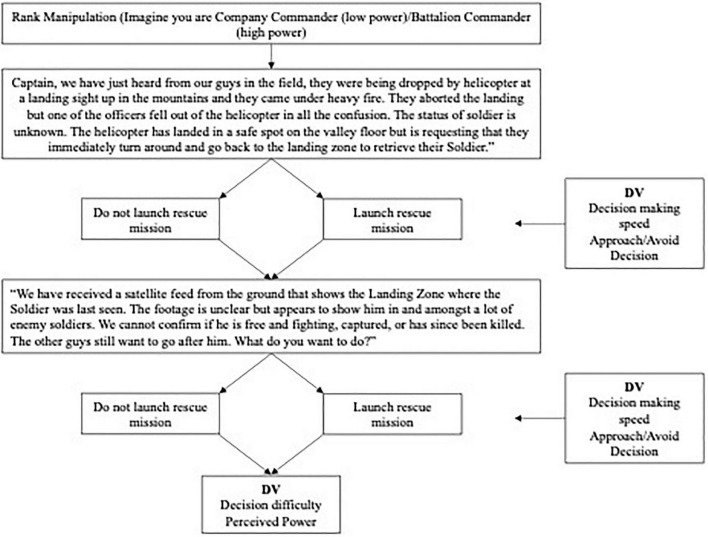
Study flow with sample rank manipulation.

Participants completed the Least-worst Uncertain Choice Inventory for Emergency Responses (LUCIFER; see Shortland et al., 2020a,b). LUCIFER adopts a two-alternative forced choice (2AFC) approach in which the participant faces a range of least-worst scenarios. Each LUCIFER scenario was developed from critical decision method (CDM) interviews with active service members of armed forces, emergency service responders, and members of the police force to ensure validity (Shortland et al., 2019; Shortland and Alison, 2020). The version of LUCIFER used in this study consisted of eight scenarios (16 total decisions). Each 2AFC choice in LUCIFER represents one approach-oriented decision (i.e., an action that makes a positive impact on the goal progress) and one avoidant-oriented decision (i.e., not taking any action that could result in harming others). After each scenario, participants were asked about their reactions to the decision-making process including perceived difficulty and perceived power. The LUCIFER study flow is presented in Figure 1. Following completion of the eight decision scenarios, participants were asked to complete a decision-making style scale.

#### Power manipulation

There exist multiple forms of social hierarchy in the U.S. Army, both formal and informal. However, for the purposes of this study, we refer to the social power by a soldier’s formal rank given that Army soldiers’ power increases with their rank. Thus, in the present study, power was manipulated by adjusting the rank of the decision-maker for each scenario. Specifically, participants were informed that they were operating (and thus making decisions) as if they were a higher rank (“Battalion Commander”) or a lower rank (“Company Commander”). Participants were informed of their rank prior to listening to the first audio clip. Following the scenario, the participant was asked to indicate how much power they perceived themselves to have in the decision scenario. Rank manipulations have previously been used to examine the effect of power (Gobel et al., 2018). Participants’ own actual rank was measured and controlled for.

### Measures

#### Within-participant time to decision

Participant time to decision scores were mean-centered. For within-participant time to decision, scores of zero indicate the average time to decision of a participant across all sessions, negative scores indicate time to decision scores below the participant’s average time to decision score, and positive scores indicate time to decision scores above the participant’s average time to decision score.

#### Within-participant power

A participant’s subjective perception of their power was assessed using a single item scale that asked the degree to which they felt they had power in the given scenario (0 = no power, 100 = high power). Scores were mean-centered so as to reduce the chances of multicollinearity (Aiken et al., 1991). This within-participant score represents changes in power, within a participant, across scenarios.

#### Within-participant confidence

Power increases self-confidence (Brinol et al., 2007; Fast et al., 2012) and previous research with the LUCIFER tool has shown that confidence is correlated with decision-making speed, difficulty and approach/avoidant tendencies (Shortland et al., 2020a,b). As such, participant confidence scores were measured and controlled for using a mean-centered within-participant confidence score. Confidence in their decision was self-reported on a scale of 0 (no confidence) to 100 (total confidence) at the end of each scenario.

#### Control variables

Based on findings from previous research with LUCIFER (Shortland et al., 2020a,b), we controlled for expertise and avoidant decision-making. Avoidant decision-making styles are associated with general avoidant decision-making (Dewberry et al., 2013); in this study we measured and controlled for trait-level tendencies to make avoidance choices using the general decision-making style avoidant subscale (DMS Avoid, Scott and Bruce, 1995). Naturalistic decision-making research places immense importance on the role of expertise in decision-making (Klein, 1998). As such, this research involved an expertise manipulation in which participants made decisions in scenarios, which were military situations (domain-specific) or non-military ones (domain-general).

## Analytic approach

A multilevel structural equation model was used to examine the nested data structure. All data analyses were conducted in R version 3.6.1 (R Core Team, 2019) using the *lme4* multilevel modeling package (Bates et al., 2020).

### Identification of data nesting structure

In accordance with previous social science research that uses structural equation modeling (SEM) frameworks to evaluate complex hypotheses with nested data, we evaluated absolute model fit to identify the ideal nesting structure for hypothesis testing (Sterba et al., 2014). This was conducted by comparing a series of intercept only models for each outcome; multilevel logistic regression for approach/avoidant decisions, and multilevel modeling for both decision time and difficulty. Both of the single nesting structure models were compared against the model with both nesting structures using a likelihood ratio test (Snijders and Bosker, 1999; Raudenbush and Bryk, 2002; Gelman and Hill, 2007) to determine the optimal nesting structure for each outcome. Nesting of avoid/approach decisions in both soldier-participants and scenarios resulted in a significant improvement of fit when compared to nesting only in soldier-participants [Δχ*^2^*(1) = 466.71, *p* < 0.01], but not when compared to nesting only in scenarios [Δχ*^2^*(1) = 0.02, *p* = 0.88]. These findings indicate that there was not significant variability in this outcome attributable to between-participant variability (i.e., no within-person nesting), but there was between scenarios (see Table 1). Due to this, subsequent analyses of avoid/approach decisions only utilized a within-scenario nesting structure. Nesting of time to decision scores in both soldier-participants and scenarios resulted in a significant improvement of fit when compared to nesting only in soldier-participants [Δχ*^2^*(1) = 145.8, *p* < 0.01] and nesting only in scenarios [Δχ*^2^*(1) = 221.77, *p* < 0.01], indicating that significant variability in this outcome is attributable to both between-participant and between-session variability (see Table 1). Due to this, subsequent analyses of time to decision utilized both a within-participant and within-scenario nesting structure. Nesting of self-rated decision difficulty scores in both soldier-participants and scenarios resulted in a significant improvement of fit when compared to nesting only in soldier-participants [Δχ*^2^*(1) = 112.63, *p* < 0.01] and nesting only in scenarios [Δχ*^2^*(1) = 607.90, *p* < 0.01], indicating that significant variability in this outcome is attributable to both between-participant and between-session variability (see Table 1). Due to this, subsequent analyses of decision difficulty utilized both a within-participant and within-scenario nesting structure.

**TABLE 1:** Participant-soldier, and scenario nesting models.

Model	Outcome	Nesting variable(s)	ICC
P	Avoid/approach	Participant-soldier	0%
S	Avoid/approach	Scenario	15.90%
PS	Avoid/approach	Participant-soldier	0.40%
		Scenario	15.90%
P	Time to decision	Participant-soldier	11.90%
S	Time to decision	Scenario	4.50%
PS	Time to decision	Participant-soldier	12.10%
		Scenario	4.50%
P	Decision difficulty	Participant-soldier	23.30%
S	Decision difficulty	Scenario	3.10%
PS	Decision difficulty	Participant-soldier	23.40%
		Scenario	3.10%

### Identification of optimum predictive model

Results showed that the baseline model (Model B) of avoid/approach decisions fit the data significantly worse [Δχ*^2^*(6) = 89.54, *p* < 0.01] than the model including interactions with scenario type (Model M), but did not differ significantly [Δχ*^2^*(6) = 6.62, *p* = 0.36] from the model including interactions with the rank manipulation (Model R). Thus, Model M was identified as the optimum model for avoid/approach decisions (see Table 2). Similarly, the baseline model (Model B) of time to decision fit the data significantly worse [Δχ*^2^*(6) = 73.36, *p* < 0.01] than the model including interactions with scenario type (Model M), but did not differ significantly [Δχ*^2^*(6) = 5.82, *p* = 0.44] from the model including interactions with the rank manipulation (Model R). Thus, Model M was identified as the optimum model for time to decision (see Table 3).

**TABLE 2:** Predictive model for approach/avoid decisions.

Predictor	Estimate	Standard error	*p*-value	Odds ratio
Intercept	0.74	0.5	0.14	2.10
Rank	0.12	0.12	0.34	1.13
Military	–1.11	0.64	0.08	0.33
Phase	–0.64	0.12	<0.01	0.53
Confidence	–0.01	0.01	0.17	0.99
Power	–0.01	0.004	0.17	0.99
DMS avoid	–0.02	0.02	0.19	0.98
Time decision	–0.08	0.01	<0.01	0.93
Military: Rank	–0.07	0.15	0.65	0.93
Military: Phase	1.04	0.15	<0.01	2.83
Military: Confidence	0.01	0.006	0.03	1.01
Military: Power	0.01	0.005	<0.01	1.01
military: DMS avoid	0.01	0.02	0.78	1.01
Military: Time decision	0.08	0.02	<0.01	1.08

**TABLE 3:** Predictive model for decision-making time.

Predictor	Estimate	Standard error	*p*-value
Intercept	5.87	1.09	<0.01
Rank	0.18	0.31	0.56
Military	–1.96	1.26	0.15
Phase	–1.00	0.30	<0.01
Confidence	–0.07	0.01	<0.01
Power	–0.02	0.01	0.15
DMS avoid	0.06	0.05	0.30
Avoid (vs. approach)	–2.08	0.34	<0.01
Military: Rank	–0.15	0.40	0.71
Military: Phase	2.55	0.38	<0.01
Military: confidence	0.004	0.02	0.81
Military: power	0.003	0.01	0.79
Military: DMS avoid	–0.06	0.05	0.20
Military: avoid (vs. approach)	2.25	0.42	<0.01

However, the baseline model (Model B) of decision difficulty fit the data significantly worse than both the model including interactions with scenario type [Model M; Δχ*^2^*(6) = 14.50, *p* = 0.02] and the model including interactions with rank manipulation [Model R; Δχ*^2^*(6) = 20.69, *p* < 0.01]. Subsequent comparisons found that Model R did not significantly differ from the model including both scenario type and rank manipulation interactions [Model RM; Δχ*^2^*(5) = 7.70, *p* = 0.17], but that Model M did significantly differ from Model RM with respect to model fit [Δχ*^2^*(5) = 13.89, *p* = 0.02]. Thus, Model R was identified as the optimum model for decision difficulty (see Table 4).

**TABLE 4:** Predictive model for decision difficulty.

Predictor	Estimate	Standard error	*p*-value
Intercept	14.15	0.92	<0.01
Rank	1.31	0.66	0.05
Military	1.11	0.67	0.14
Confidence	–0.09	0.01	<0.01
Power	–0.02	0.01	<0.01
DMS avoid	0.1	0.07	0.13
Avoid (vs. approach)	–0.03	0.27	0.90
Time decision	0.09	0.02	<0.01
Rank: military	–0.57	0.41	0.16
Rank: confidence	0.02	0.01	0.12
Rank: power	0.03	0.01	<0.01
Rank: DMS avoid	–0.1	0.05	<0.01
Rank: avoid (vs. approach)	–0.11	0.38	0.78
Rank: time decisions	–0.03	0.03	0.40

## Results

Descriptive statistics and correlations are presented in Tables 5, 6.

**TABLE 5:** Descriptive statistics for total sample.

Variable	Mean	St. deviation
**Scenario level variables (N = 3,728)**		
Decision time (DT)	6.10	6.83
Decision difficulty (DD)	15.87	6.65
Confidence	82.58	19.64
Power	78.57	22.82
**Individual level variables (N = 234)**		
Avoid/Approach score	7.53	1.88
Age	31.81	5.99
DMS avoid	10.69	3.82

	**N**	**%**

Gender (Ref = female)		
Male (Yes = 1)	122	52.14
**Scenario level variables (N = 8)**		
Military scenarios	5	

**TABLE 6:** Correlations between input variables.

Variable	1	2	3	4	5	6	7	8	9
1. Decision time	–								
2. Decision difficulty	0.130***	–							
3. Confidence	−0.161***	−0.261***	–						
4. Power	−0.067***	−0.133***	0.475***	–					
5. Avoid/approach score	0.005	0.001	0.029	0.011	–				
6. Age	0.078***	0.045**	0.097***	0.043**	0.016	–			
7. DMS avoid score	0.003	0.027	−0.114***	–0.028	−0.034*	–0.026	–		
8. Gender	0.010	−0.087***	0.220***	0.148***	0.008	0.182***	–0.026	–	
9. Military scenario	0.009	0.093***	−0.124***	−0.078***	−0.123***	0.000	0.000	0.000	–

*p < 0.05; **p < 0.01; ***p < 0.001.

### Power manipulation check

In seven of the eight scenarios, rank manipulation led to increased perceived power. A between group ANOVA was used to assess the effect of rank manipulation on perceived power. In four of the eight scenarios, participants who were assigned the higher rank reported significantly higher levels of perceived power (see Table 7). As such, the power manipulation was effective in that as participant rank increased, the subjective perceptions of the subject’s own power also increased.

**TABLE 7:** Group differences for in perceived power by rank manipulation.

	Scenario type	Rank A (lower)	Rank B (higher)	Mean difference (*p-*value*)*
**Scenario 1** (*n*_a_ = 108) (*n*_b_ = 126)	Military (domain-specific)	76.48 (23.56)	80.35 (20.84)	3.87 (*p* = 0.188)
**Scenario 2** (*n*_a_ = 109) (*n*_b_ = 125)	Military (domain-specific)	65.14 (27.62)	73.62 (25.26)	8.48 (*p* = 0.016)*
**Scenario 3** (*n*_a_ = 115) (*n*_b_ = 119)	Non-military (domain-general)	76.17 (24.00)	82.87 (18.41)	6.70 (*p* = 0.018)*
**Scenario 4** (*n*_a_ = 126) (*n*_b_ = 108)	Non-military (domain-general)	79.13 (21.38)	85.48 (18.55)	6.35 (*p* = 0.016)*
**Scenario 5** (*n*_a_ = 115) (*n*_b_ = 119)	Military (domain-specific)	76.20 (23.89)	82.58 (20.17)	6.38 (*p* = 0.027)*
**Scenario 6** (*n*_a_ = 115) (*n*_b_ = 119)	Military (domain-specific)	79.60 (20.41)	78.39 (23.83)	1.21 (*p* = 0.676)
**Scenario 7** (*n*_a_ = 132) (*n*_b_ = 102)	Non-military (domain-general)	80.33 (25.29)	80.72 (22.67)	0.39 (*p* = 0.903)
**Scenario 8** (*n*_a_ = 119) (*n*_b_ = 115)	Military (domain-specific)	78.24 (22.09)	78.77 (21.11)	0.53 (*p* = 0.849)

*p < 0.05; **p < 0.01; ***p < 0.001.

### Decision-making speed

We hypothesized that greater subjective perception of power will be positively related to the speed it takes to commit to a course of action when making a least-worst decision. The identified model indicated there were two significant interactions between scenario type (military vs. non-military) and other predictors. Avoid (vs. approach) decisions interacted with scenario type (*B* = 2.25, *SE* = 0.42, *p* < 0.01), such that in non-military scenarios making an avoid decision was associated with faster decision time (*B* = –2.08, *SE* = 0.34, *p* < 0.01), whereas in military scenarios decision time did not differ significantly as a function of the decision type (avoid vs. approach; *B* = 0.18, *SE* = 0.26, *p* = 0.48). In addition to these interaction effects, a main effect of within-subject confidence was found, whereby greater perceived within-subject confidence predicted faster decision time (*B* = –0.07, *SE* = 0.01, *p* < 0.01) across all decisions. Based on these results we find support for hypothesis 1.

### Decision difficulty

We hypothesized that greater perceived power will be positively related to the perceived difficulty of making a least-worst decision. The identified model indicated there were two significant interactions between the rank manipulation and other predictors. Specifically, rank manipulation had a significant interaction with within-participant power (*B* = 0.03, *SE* = 0.01, *p* = 0.03) participants were assigned a lower rank, their ratings of decision difficult increased when they perceived higher power (*B* = –0.02, *SE* = 0.01, *p* < 0.01) but this relationship did not hold when they were in assigned higher ranking scenarios (*B* = 0.01, *SE* = 0.01, *p* = 0.53). Additionally, there was an interaction between the rank manipulation and participants’ DMS Avoid scale scores (*B* = –0.10, *SE* = 0.38, *p* = 0.04), suggesting that the relationship between DMS Avoid scale scores and decision difficulty differ significantly as a function of the rank manipulation. However, DMS Avoid scale scores were not significantly related to decision difficulty scores for either the lower rank (*B* = 0.10, *SE* = 0.07, *p* = 0.13) or upper rank scenarios (*B* = –0.01, *SE* = 0.01, *p* = 0.94). In addition to these interaction effects, we found several main effects. Specifically, time to decision was found to positively predict decision difficulty (*B* = 0.09, *SE* = 0.02, *p* < 0.01), while within-subject confidence was found to negatively predict decision difficulty (*B* = –0.09, *SE* = 0.01, *p* < 0.01). Finally, our results showed a main effect of rank manipulation such that soldier-participants reported greater perceived difficulty when in assigned a higher rank (vs. a lower rank; *B* = 1.31, *SE* = 0.66, *p* = 0.048). These findings provide support for hypothesis 2.

### Avoid/approach decision choice

We hypothesized that increased perception of power will be negatively related to the tendency to make approach-orientated decisions when making a least-worst decision. The identified model indicated there were several significant interactions between scenario type (military vs. non-military) and predictors. Scenario phase (scenario decision 2 vs. decision 1) had a significant interaction with scenario type (*B* = 1.04, *SE* = 0.15, *p* < 0.01, *OR* = 1.01) such that in non-military scenarios participants were less likely to make an avoid decision during the second scenario prompt than the first (*B* = –0.64, *SE* = 0.12, *p* < 0.01, *OR* = 0.53), but in military scenarios participants were more likely to make an avoid decision during the second scenario prompt than the first (*B* = 0.40, *SE* = 0.09, *p* < 0.01, *OR* = 1.50). Within-participant power also had a significant interaction with scenario type (*B* = 0.01, *SE* = 0.005, *p* < 0.01, *OR* = 1.01) such that in non-military scenarios there was no effect of within-participant power (*B* = –0.006, *SE* = 0.004, *p* = 0.17, *OR* = 0.99), but that in military scenarios greater perceived within-participant power predicted increased likelihood of making an avoidant decision (*B* = 0.007, *SE* = 0.003, *p* < 0.01, *OR* = 1.01). Similarly, within-participant time to decision had a significant interaction with scenario type (*B* = 0.08, *SE* = 0.02, *p* < 0.01, *OR* = 1.08), such that in non-military scenarios when participants took longer to make a decision they were significantly less likely to make an avoidant decision (*B* = –0.08, *SE* = 0.01, *p* < 0.01, *OR* = 0.92), but there was no significant relation between time to make a decision and likelihood of making an avoidant (vs. approach) decision for military scenarios (*B* = 0.004, *SE* = 0.008, *p* = 0.65, *OR* = 1.00). While a significant interaction between within-participant confidence and scenario type (*B* = 0.01, *SE* = 0.006, *p* = 0.03, *OR* = 1.01) was identified, there were no significant effects of within-participant confidence as a predictor of the likelihood of making an avoid (vs. approach) decision in either non-military (*B* = –0.007, *SE* = 0.005, *p* = 0.17, *OR* = 0.99) or military scenarios (*B* = 0.006, *SE* = 0.003, *p* = 0.07, *OR* = 1.01). These findings provide support for hypothesis 3.

## Discussion

Understanding the relationship between power and decision-making tendencies is critically important given that those in positions of power often face high stakes decisions that involve juggling approach/avoidance courses of action between the lesser of two evil outcomes. The approach-inhibition theory of social power holds that elevated power activates approach-related tendencies, leading to decisiveness and action orientation. However, naturalistic decision-making research has often reported that increased power often has the opposite effect and causes more avoidant decision-making. Thus, while the approach-inhibition theory posits a single, linear relationship between power and BAS activation, real-world evidence seems to imply that, in some conditions, power can lead to tendencies to avoid (and thus BIS activation). Accordingly, this study sought to explore the effect of social power on least-worst decision-making. In line with our hypotheses, and the wider naturalistic decision-making findings, this research found that increases in perceived power were associated with avoidant choices and increased difficulty. To the author’s knowledge, this is the first experimental study to show that the theory of approach-inhibition may be missing the potential relationship between power and avoidance. This theoretical extension of the current linear conceptualization of power and BAS is critically important given the need to fully understand how the environment may impact on decision-maker in high-uncertainty situations.

While the domain of laboratory-based research on decision-making has widely supported the assertion that power drives action (Guinote, 2007; Pike and Galinsky, 2020), findings from naturalistic decision-making research perhaps explains why this is not always the case. In a simulated study of counter-terrorism police decision-making, the presence of accountability (often associated with those in power; Rus et al., 2012) caused people to shift from approach goals (save lives) to an avoidant goal (save self and own career; van den Heuvel et al., 2014). In fact, the study of organizational accountability has often found that accountability affects the choices that an individual makes and their ability to commit to a decision (van den Heuvel et al., 2012; Shortland et al., 2019). Accordingly, police officers’ performance decreased when they felt accountable for their actions (Waring et al., 2013). This was suggested to stem from an increase in cognitive load due to additional concerns associated with being accountable. Accountability can also increase risk aversion, encouraging decisions that protect oneself and the consequences of one’s actions, rather than a commitment to decisions that are the best action for the major incident (Alison et al., 2015a). This in turn can create redundant deliberation by competing with the desire to make a positive impact on the situation (Power and Alison, 2017). Such views of accountability would explain why increased perceived power was associated with tendencies to make avoidant choices. This view of accountability and rank may also explain the finding that higher rank led to lower perceived power in that higher ranks were viewed as being more constrained and associated with higher levels of accountability, and thus, contra-logically, individuals felt less powerful.

The finding that participants did not show a marked improvement in approach tendencies, speed, or difficulty, when facing domain-specific decisions potentially stems from the notion that least-worst decisions, by nature, violate many of the contemporary models of expert decision-making. Principal features of expert decision-making are improved mental models and an increased ability to pattern-match (Allard et al., 1980). Least-worst decisions are often associated with “no analogies,” and thus, individuals have no previous mental models that can be readily applied to the situations. Those who recall least-worst decisions often emphasize that they were “new” or “novel” (Shortland et al., 2019, 2020a; Shortland and Alison, 2020). This perhaps explains why, overall, domain expertise had little effect on decision-making. That said, participants were faster to make avoidant choices in situations for which they had no expertise. One view of this is that, in those situations in which participants had domain-expertise, they were better able to process the context of the decisions, and indeed the future outcomes or a choice (“what will happen if”). This may have led to a slowing of the decision-making process because while participants were equally avoidant in both types of scenario, their increased understanding of the context of a military scenario (that they have domain expertise in) added additional workload.

Beyond supporting the elsewhere observed effect of power on avoidance, these findings raise questions about how we think about decision-making. For example, while naturalistic research has often focused on the processes that underpin decision-making, the role of motivation processes in decision-making is less often integrated. This study questions the degree to which the avoidance and approach tendencies identified elsewhere (Power and Alison, 2017), can be conceptualized in the approach and avoidance tendencies highlighted in Gray’s (1987) RST. In this case, we can think of processes such as decision inertia as a manifestation of BIS processing, which has interesting implications for identifying the neurological, and state and trait personality factors that may play a role in the emergence of this behavior.

### Limitations

More open research methodologies (such as LUCIFER) allow for the identification of the wider exogenous and endogenous pressures (e.g., accountability, trust and role confusion) that would, arguably, not manifest in more “closed” decision-making tasks (termed “immersive simulated learning environments”; see Alison et al., 2013). With that said, in this study, it is viable that the engagement and immersion of the participant increased the role of unmeasured variables to the interpretation. For example, while the context of leadership could have increased subjective perceptions of accountability, it is also possible that the audio immersion activated the BIS or fight, flight, freeze system (BIS/FFFS; McNaughton, 2004). While we do not advocate the use of research methodologies with lower fidelity, increasing the fidelity of the methodology (a strength of LUCIFER) does increase the potential extraneous variables that may have unintended, and unforeseen influences on behavior. As such, we encourage future research to measure wider organizational and ecological variables, such as a culture of accountability, that may play an important role in decision-making.

## Conclusion and future directions

We increasingly ask for more accountability in those with power (e.g., police, military, and world leaders; Rossler, 2019; Crayne and Medeiros, 2021). These findings emphasize the need to explore the immense importance of wider exogenous factors (such as accountability) when theorizing processes as complex as decision-making under uncertainty and the effect of social power. While it is a staple finding in the field that power increases approach motivations and improves decision-making (Kang et al., 2015), this study questioned the universal positive and approach effects of power and instead found that those with power were more avoidant, and indeed found decisions harder, especially when making decisions they had experience with (domain-specific). This study thus challenges the linear conceptualization of approach-inhibition theories of social power and proposes that, under certain conditions, power can lead to FFFS/BIS activation and avoidance tendencies. This advances the need for future research that explores the conditions in which power leads to avoid, rather than approach tendencies.

There are also several further questions to be explored. For example, recent research has shown that individual differences in personality characteristics (e.g., maximization) impact the degree to which decision-making is impacted by interventions (Shortland et al., 2021). As such, it is viable to propose that individual differences in certain key personality traits may moderate the effect of power on avoidance. Furthermore, it is also important to acknowledge the degree to which the relationship between power and avoidance may be cyclical. That is avoidance may lead to a decrease in how others in the organization perceive the individual, decreasing their power further and leading to more avoidance. Finally, it is important to consider how the role of power integrates with other known sources of avoidance. For example, previous research has highlighted the role of organizational culture and “career fear” (van den Heuvel et al., 2013). While this study highlights an important individual-level contribution to the tendency to make avoidant decisions, naturalistic research makes it evidently clear that avoidance is the result of a range of influences as the individual, cultural and environmental, level, and future research needs to further explore the interactions of these factors. That said, and despite these further theoretical questions, this research provided a critical addition to our understanding of the many (and not always positive) impacts of power on decision-making.

## Data availability statement

The raw data supporting the conclusions of this article will be made available by the authors, without undue reservation.

## Ethics statement

The studies involving human participants were reviewed and approved by the University of Massachusetts Lowell. The patients/participants provided their written informed consent to participate in this study.

## Author contributions

NS, MM, and NB: conceptualization, methodology, data curation, and writing original draft. LT: writing original draft, data curation, and formal analysis. LA: conceptualization, methodology, supervision, and review. JG: statistics. PM and CS: writing and editing and theorizing. All authors contributed to the article and approved the submitted version.
